# A Case of Erythema Nodosum in a 20-Year-Old Female During the Postpartum Period

**DOI:** 10.7759/cureus.58526

**Published:** 2024-04-18

**Authors:** Martin Nguyen, Christopher Gross, Seo Young Huh, Abigail Frank

**Affiliations:** 1 Medical School, West Virginia School of Osteopathic Medicine, Lewisburg, USA; 2 Family Medicine, West Virginia School of Osteopathic Medicine, Lewisburg, USA

**Keywords:** sarcoidosis, tuberculosis, panniculitis, postpartum period, pregnancy, erythema nodosum

## Abstract

Erythema nodosum (EN) is the most common form of panniculitis and occurs in about one in 100,000 people. EN typically presents as an eruption of tender, erythematous nodules on the anterior aspect of the legs, although the face, trunk, and arms can also be involved. While the majority of cases are idiopathic, a subset of cases occurs in association with various triggers, including infections, medications, tumors, and autoimmune diseases. Rarely can EN develop in relation to pregnancy, which is thought to provide a physiologic background that favors its development. While pregnancy has been associated with EN in a minority of cases, currently, there is a limited amount of data suggesting that EN can develop in the late postpartum period. Herein, we present a case of a 20-year-old female with a six-week history of painful lesions on her lower extremities. A physical exam revealed multiple tender, erythematous nodules on the anterior aspect of the lower extremities, spanning from the knees to the toes. Laboratory workup showed no other identified triggers of EN in our patient besides pregnancy. Management of EN in our patient involved a low dose, six-day course of prednisone (initial dose of 15 mg/day) and ibuprofen for one week, leading to symptomatic improvement. Our case emphasizes the possibility of EN presenting in the late postpartum period. This case underscores the importance of considering EN in the differential diagnoses for women presenting with compatible lesions postpartum.

## Introduction

Adipose is a specialized form of connective tissue that both stores energy and provides protection against trauma. The panniculus adiposus, also known as the subcutaneous fat, is a layer of adipose tissue located beneath the skin. Panniculitis is a rare condition characterized by inflammation involving subcutaneous fat. Erythema nodosum (EN) is the most common form of panniculitis. It is characterized by an acute onset of erythematous tender nodules and plaques, mainly on the pretibial area of lower limbs [1]. The exact pathogenesis of EN remains unclear. EN is typically self-limiting and spontaneously resolves without ulceration, scarring, or atrophy [2]. EN is a diagnosis of exclusion and thus requires extensive workup to rule out other potential differential diagnoses. EN is primarily idiopathic, but it can be one of the first signs of systemic diseases. Associated triggers include drugs, infection, autoimmune disorders, pregnancy, and malignancy. Pregnancy is thought to play a key role in EN and accounts for about 2% of EN cases [3]. EN in pregnancy often resolves by the third trimester [3]. In addition, EN during the postpartum period is relatively rare. Morris et al. [4] reported the first case report in 2018. Herein, we present a case of EN in a 20-year-old female who presented in the postpartum period without additional identifiable triggers.

This article was previously presented as a poster at the 2024 West Virginia School of Osteopathic Medicine (WVSOM) Research Poster Competition on March 5, 2024.

## Case presentation

**Figure 1:**
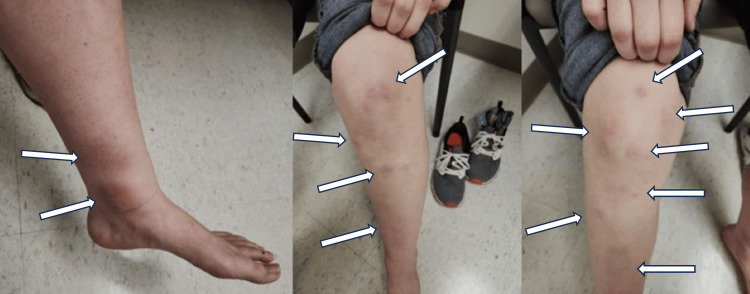
Multiple erythematous nodules on the bilateral knees, legs, and ankles.

A 20-year-old female presented to a rural health clinic with a six-week history of painful nodules in her bilateral lower extremities. A physical exam revealed multiple tender, erythematous nodules present bilaterally on the knees, legs, ankles, and great toes (Figure 1). Medical history included a term singleton delivery approximately 10 weeks before the onset of the painful nodules. Laboratory workup revealed both a negative rheumatoid factor (RF) and cyclic citrullinated peptide antibody (CCP), mildly elevated ESR (26 mm/h, normal < 20 mm/h). The patient did not present with symptoms suggesting a respiratory infection (e.g., fever, fatigue, and weight loss). The patient was also not indicated for antistreptolysin O (ASO) titer due to the lack of suggestive findings (e.g., fever, sore throat, and oral erythema). Further testing showed a negative tuberculin test. Given the presentation of our patient, as well as the lack of notable other inciting factors, a diagnosis of EN was made. Afterward, our patient was prescribed a short course of low-dose oral prednisone and ibuprofen. The initial dose of prednisone was 15 mg/day and then was tapered for six days. Ibuprofen 400 mg tablets were indicated for use as needed. Upon follow-up appointment two weeks later, the patient stated less pain and objective improvement in the size and quantity of nodules. Although relatively rare, our case displays the importance of the primary care provider’s ability to recognize and effectively treat EN.

## Discussion

EN can occur at any age with the majority of cases occurring at 20-30 years of age, possibly related to the higher incidence of sarcoidosis in this age group [5]. Multiple studies have demonstrated that EN shows a strong female predilection, with females tending to be affected three to six times more frequently than males [6,7]. EN has been associated with drugs, infections, tumors, and autoimmune diseases, although many cases are idiopathic (Table 1). The incidence of these etiologies mainly depends on ethnic and geographic differences, ranging from 37% to 72% [1,8,9]. In a retrospective study with 106 patients in Spain, idiopathic cases represented 36.8% of all cases [6]. The study was conducted during a 10-year period (1988-1997), and all the cases were biopsy-proven [6]. The incidence rate was 52/1,000,000 with a female predominance (female-to-male ratio = 3.27:1). Among secondary EN cases, sarcoidosis was the most common identifiable cause [6]. However, as a group, infections are the most common cause of secondary EN with 33.3% of cases, with Group A Streptococcus (GAS) and tuberculosis as the most common pathogens (6.9% and 4.9%, respectively). Other etiologies include a history of non-streptococcal upper respiratory infections (URIs), with or without the use of antibiotics (6.9% and 12.7%, respectively). In another retrospective study by Mert et al. [7], there were 50 cases of EN in 10 years in a Turkish hospital. Females were predominantly affected (female-to-male ratio = 6:1), and 46% of all cases were idiopathic. Among secondary EN cases, the most common causes included primary tuberculosis (TB) (18%), post-streptococcal infections (16%), inflammatory bowel disease (IBD) (4%), Behçet’s disease (2%), and pregnancy (2%) (Table 1). One notable feature was that the nodules tended to relapse every year in 74% of idiopathic EN patients, but this occurred in only one case of secondary EN (Behçet’s disease) [7]. This highlights the importance of treating secondary causes of EN whenever possible.

**Table 1:** Common etiologies of erythema nodosum. TB: tuberculosis, EN: erythema nodosum Source: [6,7,10]

	Garcia-Porrua et al. [6] (N = 106) (%)	Mert et al. [7] (N = 50) (%)	Cribier et al. [10] (N = 129) (%)
Country	Spain	Turkey	France
Idiopathic EN	34.3	46	55
Secondary EN	65.7	54	45
Primary TB	4.9	18	0.8
Poststreptococcal	6.9	16	28
Sarcoidosis	21.6	12	10.8
Drugs	2.9	N/A	
Ulcerative colitis	1	2	N/A
Crohn’s disease	2	2	N/A
Behçet’s disease	2	2	N/A
Pregnancy	N/A	2	N/A
Sweet’s disease	2	N/A	N/A

EN may be the first sign of a systemic or infectious disease. Factors pointing toward a secondary cause of EN included fever, cough, sore throat, diarrhea, abnormal chest X-ray, leukocytosis, increased erythrocyte sedimentation rate (ESR > 50 mm/h), increased C-reactive protein levels, previous history of URI, synovitis, or positive tuberculin test [6,7].

EN is a painful disorder of the subcutaneous fat, which, in adults, has a predilection for females [11]. Although there is data pointing to a correlation between the development of EN in pregnancy - primarily in the first trimester - there is a paucity of literature related to the development of pregnancy in the postpartum period [4]. Numerous theories pertaining to the physiological, immunological, anatomical, chemical, and hormonal changes that occur in the pre/post-partum period have attempted to provide a pathophysiological explanation for the development of EN. Despite various attempts to explain the development of EN, a definitive cause has yet to be identified, with most cases developing idiopathically.

The reason pregnancy may trigger EN is currently not completely understood. However, it seems to be related to hormonal changes during pregnancy [12]. This correlation is further supported by the fact that oral contraceptive pills (OCPs) have been associated with EN development [12,13]. Furthermore, Papagrigoraki et al. [14] have shown that sex hormones, including OCPs, were the most common drugs to trigger EN. Another theory suggests that pregnancy stimulates immune complex deposition in subcutaneous fat [3,15]. Cribier et al. [10] suggested that estrogen can facilitate EN development, while other authors emphasized that progesterone or the ratio of progesterone to estrogen is more important than estrogen alone in EN eruption [2,15]. During pregnancy, EN tends to occur in the first trimester. No matter what the link between EN and pregnancy could be, it is well-established that pregnancy modulates the immune system and potentially contributes to the triggering of EN.

Mechanical factors may also contribute to the increased risk of EN during pregnancy. As mentioned above, EN primarily occurs in the anterior shin of the lower limbs. Some authors have suggested that the effects of gravity, a poor lymphatic system, and a lack of muscles in the shin make it more difficult for the body to manage local inflammation [15]. Moreover, increased edema in pregnancy may worsen the problem. During the postpartum period, it is postulated that hormonal changes from pregnancy to lactation make women more susceptible to EN [16].

Typical clinical features include a sudden abruption of tender, symmetrical, erythematous nodules often located in the lower extremities. The size of nodules often ranges from 1 to 5 cm and is typically bilaterally distributed [2]. Nodules may be confluent and lead to the formation of erythematous plaques. Initially, these nodules have a bright red color and are raised above the skin. After a few days, the lesions become flat and the color evolves to purplish red. Late lesions may show a yellowish or greenish hue that resembles a deep bruise [2]. EN may be accompanied by non-specific symptoms, such as fever, fatigue, malaise, arthralgia, headache, cough, or diarrhea. Symptoms vary depending on the secondary cause, but these non-specific symptoms may also be present with idiopathic EN. Moreover, recurrences are not uncommon. Some clinical variants of EN have been described under different names, including EN migrans, subacute nodular migratory panniculitis, and chronic EN. However, most authors agree that these variations should be included in the spectrum of EN [17].

A cost-effective approach to reach an accurate diagnosis of EN is required due to an extensive list of possible etiologies. Initial laboratory evaluation should include a complete blood count (CBC), ESR, ASO titer, urinalysis, throat culture, tuberculin test, and chest X-ray [2]. In this patient, she was indicated to have CBC, ESR, urinalysis, ANA, anti-CCP, and tuberculin test. Both ANA and anti-CCP were negative, suggesting that rheumatoid arthritis (RA) or systemic lupus erythematosus (SLE) as less likely etiologies in this case. Our patient also had a negative tuberculin test. Although she did not have a chest X-ray during her visit, the lack of respiratory symptoms and systemic symptoms (e.g., fever, fatigue, weight loss) coupled with a negative tuberculin test did not suggest sarcoidosis or primary tuberculosis as a potential secondary cause of EN. In addition, ASO titer was not indicated in this patient due to the lack of suggestive findings (e.g., fever, sore throat, and oral erythema). Although ESR was mildly elevated, we did not think that this may suggest a secondary cause. According to Mert et al. [7], ESR > 50 mm/h or CRP > 6 times of the upper normal limit would suggest a secondary cause of EN. All the other laboratory tests were unremarkable.

EN is a self-limiting condition with symptoms often resolving within a few weeks (three to six weeks) [18]. Management includes treating underlying identifiable conditions and symptom control. Conservative treatment with oral non-steroidal anti-inflammatory drugs (NSAIDs), as well as short-term administration of systemic steroids, have been documented as effective interventions [11]. During pregnancy, bed rest and compression bandages may be indicated. Non-steroidal anti-inflammatory drugs, including aspirin, can be used during the postpartum period, but they should be avoided during pregnancy [15]. If the symptoms are NSAID-refractory, steroid treatment may be considered in the second and third trimesters and the postpartum period. Oral prednisone 40 mg/day has been successfully utilized in severe cases [1]. Infections and malignancy should be ruled out before corticosteroids are initiated. Minocycline 100 mg twice daily has also been considered for chronic EN. Tumor necrosis factor alpha (TNF-α) inhibitors, such as etanercept, have also been used in resistant cases. Other options include potassium iodide, colchicine, and dapsone. However, these drugs are not recommended during pregnancy or for breastfeeding women due to their adverse effects on fetuses and neonates [19].

## Conclusions

Although the association between pregnancy and EN has been established, it is relatively rare to see EN during the postpartum period. Our case presented with classic dermatological findings including multiple erythematous nodules on both knees, legs, ankles, and toes. This case serves as a reminder that EN should be included in differential diagnoses in women during the postpartum period presenting with compatible cutaneous findings.
